# Reduced expression of FILIP1L, a novel WNT pathway inhibitor, is associated with poor survival, progression and chemoresistance in ovarian cancer

**DOI:** 10.18632/oncotarget.12784

**Published:** 2016-10-20

**Authors:** Mijung Kwon, Jae-Hoon Kim, Yevangelina Rybak, Alex Luna, Chel Hun Choi, Joon-Yong Chung, Stephen M. Hewitt, Asha Adem, Elizabeth Tubridy, Juan Lin, Steven K. Libutti

**Affiliations:** ^1^ Department of Surgery, Albert Einstein College of Medicine, Bronx, NY 10461, USA; ^2^ Division of Biostatistics, Department of Epidemiology and Population Health, Albert Einstein College of Medicine, Bronx, NY 10461, USA; ^3^ Department of Obstetrics and Gynecology, Gangnam Severance Hospital, Yonsei University College of Medicine, Seoul 135-720, Korea; ^4^ Institute of Women's Life Medical Science, Yonsei University College of Medicine, Seoul 135-720, Korea; ^5^ Department of Obstetrics and Gynecology, Samsung Medical Center, Sungkyunkwan University School of Medicine, Seoul 135-710, Korea; ^6^ Experimental Pathology Laboratory, Laboratory of Pathology, Center for Cancer Research, National Cancer Institute, National Institutes of Health, Bethesda, MD 20892, USA

**Keywords:** ovarian cancer, prognosis, metastasis, chemoresistance, WNT

## Abstract

Filamin A interacting protein 1-like (FILIP1L) is an inhibitor of the canonical WNT pathway. WNT/β-catenin signaling and its downstream pathway, epithelial-to-mesenchymal transition (EMT), play a key role in ovarian cancer metastasis and chemoresistance. To study the clinical implications of FILIP1L in regulating the WNT/β-catenin pathway, the expression of FILIP1L, β-catenin, SNAIL and SLUG was analyzed by immunohistochemistry on tissue microarrays of 369 ovarian samples ranging from normal to metastatic. In addition, the results were validated in mouse model and *in vitro* cell culture. In the present study, we demonstrated that FILIP1L expression was inversely correlated with poor prognosis, stage and chemoresistance in ovarian cancer. Notably, low FILIP1L expression was independent negative prognostic factor with respect to overall and disease-free survival. FILIP1L inhibited peritoneal metastases in orthotopic mouse model. FILIP1L knockdown induced chemoresistance in ovarian cancer cells and this phenotype was rescued by simultaneous knockdown of FILIP1L and SLUG, an EMT activator. We also demonstrated that FILIP1L regulates β-catenin degradation. FILIP1L co-localizes with phospho-β-catenin and increases phospho-β-catenin at the centrosomes, destined for proteosomal degradation. Finally, we showed that FILIP1L regulates EMT. Overall, these findings suggest that FILIP1L promotes β-catenin degradation and suppresses EMT, thereby inhibiting metastases and chemoresistance. Our study provides the first clinical relevance of FILIP1L in human cancer, and suggests that FILIP1L may be a novel prognostic marker for chemotherapy in ovarian cancer patients. Further, the modulation of FILIP1L expression may have the potential to be a target for cancer therapy.

## INTRODUCTION

Ovarian cancer is the most general cause of death from gynecological cancers and the fifth leading cause of death from all cancers in women [1]. Most patients are diagnosed with advanced stage metastatic disease [1]. Following surgical cytoreduction, the standard cytotoxic systemic regimens consist of platinum and a taxane, and most patients attain initial complete clinical remission [2]. The majority of them, however, will eventually have a relapse and die of their disease [2, 3]. The overall survival rate, while improved by recent advances in targeted therapy, remains poor, with the five-year survival rate below 25% for stage III-IV disease [1, 4]. Thus, a better understanding of the pathways involved in metastasis and chemoresistance is crucial for the development of more effective therapies in ovarian cancer.

There is abundant, published evidence for the central role of WNT signaling in ovarian cancer biology [5–9]. The canonical WNT signaling leads to the nuclear accumulation of β-catenin and transcriptional activation of target genes [10]. Genetic and epigenetic deregulation of various steps of WNT signaling results in metastasis and chemoresistance in ovarian cancer [7–9]. Increased β-catenin levels are commonly observed in different ovarian cancer subtypes and correlate with cancer stage and poor survival [6, 11–14].

The WNT/β-catenin signaling pathway was first described for its role in carcinogenesis and later shown to regulate epithelial-to-mesenchymal transition (EMT) during embryogenesis [15]. As a result of EMT, epithelial cells gain a pluripotent mesenchymal phenotype, featured by loss of cell adhesion, acquisition of mesenchymal markers, increased cell motility and resistance to programmed cell death [16]. In cancer, the properties of mesenchymal cells directly contribute to their enhanced invasiveness and chemoresistance [17]. Key regulators of EMT are up-regulated by WNT/β-catenin signaling, including transcription factors SNAIL, SLUG, ZEB and TWIST [16,18]. Activation of WNT/β-catenin signaling is linked to initiation of EMT in ovarian cancer cells [8, 12, 19–23].

The loss of E-cadherin, a key feature of EMT, occurs in most carcinomas, and facilitates metastasis [24]. EMT has been involved in ovarian cancer metastasis [25, 26]. However, a unique characteristic of ovarian cancer is that primary differentiated carcinomas gain E-cadherin expression compared to the mesenchymally-driven normal ovarian surface epithelium [27, 28]. Subsequent regain of mesenchymal characteristics has been observed in more advanced tumors with associated loss of E-cadherin expression during metastatic progression [28–30]. Despite the dual expression pattern of E-cadherin in ovarian cancer progression, its expression is related with longer survival in the most of studies with clinical ovarian carcinoma specimens [25, 31, 32]. Forced expression of E-cadherin results in inhibition of ovarian cancer metastasis [33]. In contrast, the expression of mesenchymal markers such as N-cadherin, SNAIL and TWIST is associated with poor prognosis [25, 34, 35]. Molecular expression profiles from clinical samples also support the role of EMT in chemoresistance [36, 37] as well as metastasis [12, 38] in ovarian cancer. Inhibiting EMT-like events is therefore a reasonable therapeutic strategy for combating ovarian cancer progression.

Filamin A interacting protein 1-like (FILIP1L) inhibits the canonical WNT pathway [39]. Its inhibition by FILIP1L results in the transcriptional down-regulation of WNT target genes, leading to the inhibition of cell invasion and metastasis [39]. We originally observed that FILIP1L was down-regulated through promoter methylation, and its down-regulation induced an invasive phenotype in various types of cancer cells including ovarian [39–42]. Modulation of FILIP1L expression in these cancer cells led to the changes in cell invasion [39–41]. We also demonstrated FILIP1L has an anti-angiogenic function that over-expression of FILIP1L in endothelial cells led to decreased cell migration and increased apoptosis, and that tumor vessel-expression of FILIP1L blocked *in vivo* tumor growth [42]. FILIP1L appears to be widely expressed in human tissues and its physiologic function is currently not known. There are five known isoforms of FILIP1L that are generated by alternative splicing; our work focuses on the most prevalent isoform 2 [40, 43, 44]. Its structural homologies and centrosomal localization suggest that FILIP1L may bind elements of the cytoskeleton and chaperone proteins to proteasomes [39, 43, 45]. However, the clinical relevance of FILIP1L down-regulation in cancer progression has yet to be addressed.

In this study, we show that FILIP1L is a marker of prognosis, stage and chemosensitivity in ovarian cancer. FILIP1L knockdown induces chemoresistance in ovarian cancer cells. Moreover, the tumor-suppressor activity of FILIP1L is exerted through its inhibition of EMT, a mechanism of metastasis and chemoresistance in ovarian cancer.

## RESULTS

### FILIP1L is a marker of prognosis, stage and chemosensitivity in ovarian cancer

**Figure 1:**
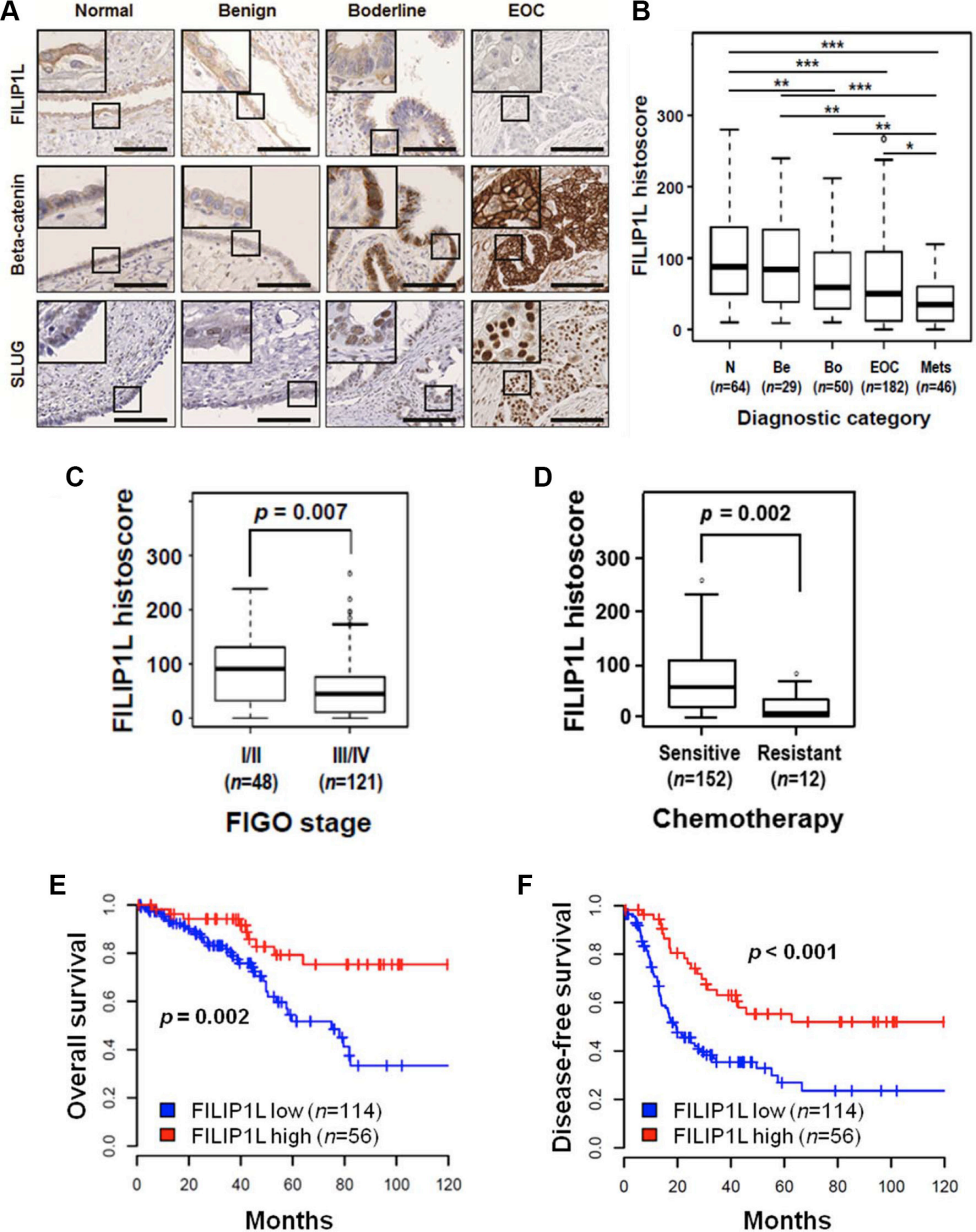
Expression of FILIP1L, β-catenin and SLUG in normal and cancerous ovarian tissue (**A**) Immunohistochemical staining of FILIP1L, β-catenin and SLUG was performed on tissue microarray of human ovarian cancer specimens. The final histoscore, ranging from 0-300, was calculated by multiplying the intensity by percentage of staining. A. Representative images of immunohistochemical staining of FILIP1L, β-catenin and SLUG in ovarian tissues from normal, benign, borderline, and carcinoma patients. High magnification images are shown in inset. Scale bar shown is 100 μm. (**B**) Expression of FILIP1L was analyzed in specimens from normal (*n* = 64), benign (*n* = 29), borderline (*n* = 50), epithelial ovarian cancer (*n* = 123 for Serous type; *n* = 59 for other histologies) and metastasis (*n* = 46). *, ** and *** indicate *P* < 0.05, *P* < 0.01 and *P* < 0.001, respectively. (**C**) FILIP1L expression was compared between specimens from FIGO stage I/II (*n* = 48) and stage III/IV (*n* = 121) ovarian cancers. (**D**) FILIP1L expression was compared between specimens from chemosensitive (*n* = 152) and chemoresistant (*n* = 12) ovarian cancer patients. Cancer patients who had recurrence within 6 months after platinum and paclitaxel chemotherapy were considered resistant. (**E–F**) Overall survival (E) and disease-free survival (F) of ovarian cancer patients who were positive for FILIP1L was analyzed in Kaplan–Meier plots. Receiver operating characteristic (ROC) analysis was used to determine histoscore cut-off values. Histoscore of ≥ 75 for FILIP1L expression was considered high.

We previously showed that FILIP1L inhibits the canonical WNT pathway by reducing nuclear β-catenin amount and β-catenin-directed transcriptional activity [39]. As described earlier, the WNT/β-catenin pathway and its downstream target pathway EMT has been implicated in ovarian cancer metastasis and chemoresistance. In particular, EMT-regulating transcription factors SLUG and SNAIL were shown to be directly associated with cisplatin and paclitaxel resistance in ovarian cancer [46, 47]. To study the clinical implications of FILIP1L in regulating the WNT/β-catenin pathway and chemoresistance, we examined the expression of FILIP1L, β-catenin, SNAIL and SLUG in ovarian cancer specimens from patients in whom clinical outcome data were available. Immunohistochemical staining on tissue microarrays of ovarian samples ranging from normal to metastatic were performed. The clinicopathological features of the study are detailed in Table 1. We showed that loss of FILIP1L expression increases with tumor progression, resulting in a significant difference between primary and metastatic ovarian cancer samples, whereas the levels of β-catenin and SLUG increase with tumor progression (Figure 1A, 1B and Supplementary Figure S1A). As shown in Supplementary Figure S2, FILIP1L expression was significantly decreased in metastatic tissues compared with primary ovarian cancer tissues from matching patients. There were no differences in these protein levels between Serous and other ovarian cancer histologies (Table 1). Further substantiating the inverse relationship between FILIP1L levels and tumor progression was the difference in its expression between early and late stage ovarian cancer (Figure 1C). Importantly, tumors that were resistant to platinum/paclitaxel combination therapy showed a significant reduction in FILIP1L expression when compared to sensitive tumors (Figure 1D). In contrast, SLUG expression was significantly higher in resistant tumors than in sensitive tumors (Supplementary Figure S1C). FILIP1L expression was correlated negatively with the expression of β-catenin and SLUG, whereas β-catenin expression was correlated positively with SLUG expression, suggesting a link between FILIP1L and the WNT/β-catenin pathway in ovarian cancer (Supplementary Figure S1D). Although expression of SNAIL increased with tumor progression, it was not different between chemo-resistant and chemo-sensitive tumors, nor was there a negative correlation with the expression of FILIP1L (data not shown; a representative image of immunohistochemical staining of SNAIL is shown in Supplementary Figure S3).

**Table 1:** Association of FILIP1L, Beta-catenin, and SLUG expression with clinicopathological characteristics in normal and neoplastic ovarian tissue samples

	No	FILIP1L		Beta–catenin		SLUG	
		Median [IQR]	*p* value	Median [IQR	*p* value	Median [IQR]	*p* value
**Diagnostic category**							
Normal	64	88 [50–142]	< 0.001	54 [30–89]	< 0.001	110 [79–173]	< 0.001
Benign	29	84 [39–140]		66 [34–100]		141 [86–195]	
Borderline	50	59 [29–105]		84 [60–128]		131 [68–183]	
Cancer	182	50 [13–109]		106 [71–150]		144 [91–183]	
Metastatic	46	35 [12–60]		100 [70–131]		224 [162–266]	
**Age**							
≤ 50	85	56 [18–123]	0.032	110 [59–149]	0.808	146 [99–180]	0.755
> 50	97	41 [7–85]		99 [74–150]		143 [83–196]	
**Histological subtype**							
Serous	123	50 [17–101]	0.914	101 [73–145]	0.531	146 [86–197]	0.647
Others	59	55 [7–119]		110 [64–170]		140 [107–165]	
**FIGO stage**							
I/II	48	91 [30–122]	0.007	101 [60–143]	0.535	136 [81–163]	0.102
III/IV	121	41 [11–76]		101 [74–150]		145 [91–199]	
**Tumor grade**							
Well + Moderate	78	51 [12–110]	0.916	110 [80–161]	0.144	143 [83–179]	0.589
Poor	91	50 [16–104]		98 [60–143]		140 [91–181]	
**CA125**							
≤ 35	24	43 [20–130]	0.238	99 [60–134]	0.632	136 [87–155]	0.290
> 35	154	51 [11–102]		106 [73–151]		145 [91–183]	
**Chemosensitivity**							
Sensitive	152	55 [16–113]	0.002	104 [70–150]	0.182	141 [90–182]	0.041
Resistant	12	5.5 [0–27]		136 [89–155]		168 [147–190]	

a Calculated only 169 cases with available information of FIGO stage and tumor grade.

b Calculated only 178 cases with available information of CA125.

c Calculated only 163 cases with available information of chemosensitivity.

Protein expression was determined through analysis of an immunohistochemically stained tissue array, as described in the materials and methods section.

We next examined the relationship between the expression levels of these proteins and patient survival. We demonstrated that high FILIP1L expression correlates with a significantly improved overall survival and disease-free survival over a ten-year period post-chemotherapy (Figure 1E, 1F). Notably, patients with low FILIP1L expression showed a median overall survival and disease-free survival of 60 and 19 months, respectively, whereas patients with high FILIP1L expression had not yet reached median overall and disease-free survival at their 120 month follow-up. When we analyzed for serous ovarian cancer patients only, we also demonstrated that high FILIP1L expression correlates with a significantly improved overall survival and disease-free survival (Supplementary Figure S4). Furthermore, patients with a combination of high FILIP1L and low β-catenin or SLUG expression had significantly longer overall survival than patients with low FILIP1L and high β-catenin or SLUG expression (Figure 1E, 1F and Supplementary Figure S1E, S1F). The Cox proportional hazards model revealed that low FILIP1L expression and a combination of low FILIP1L and high SLUG expression were independent negative prognostic factors with respect to overall survival (Table 2). For disease-free survival, low FILIP1L, high SLUG and a combination of low FILIP1L and high β-catenin or high SLUG, and of high β-catenin and high SLUG expression were independent negative prognostic factors (Table 3). Together these results support the observation that FILIP1L may be a useful tumor marker whose expression is inversely correlated with poor prognosis, stage and chemoresistance, possibly due to its role in inhibiting WNT/β-catenin pathway.

**Table 2:** Univariate and multivariate analyses of overall survival in ovarian cancer patients

	Univariate analysis		Multivariate analysis	
Variables	Hazard ratio [95% CI]	*p* value	Hazard ratio [95% CI]	*p* value
Age (> 50)	2.20 [1.17–4.13]	0.014	2.02 [1.02–3.98]	0.043
FIGO stage (III/IV)	4.72 [1.68–13.24]	0.003	3.15 [1.08–9.18]	0.036
Cell type (non-serous)	0.31 [0.13–0.73]	0.008	0.56 [0.23–1.34]	0.193
Grade (poor)	1.84 [1.00–3.39]	0.049	1.73 [0.90–3.29]	0.098
CA125 (> 35)	1.97 [0.70–5.50]	0.196	NA	
FILIP1L-	3.04 [1.46–6.34]	0.003	2.30 [1.03–5.15]	0.043
Beta-catenin+	0.72 [0.40–1.30]	0.283	NA	
SLUG+	1.80 [0.97–3.34]	0.062	NA	
FILIP1L-/Beta-catenin+	2.80 [0.91–8.63]	0.074	NA	
FILIP1L-/SLUG+	3.08 [1.27–7.47]	0.013	2.77 [1.08 – 7.07]	0.033
Beta-catenin+/SLUG+	1.36 [0.58–3.18]	0.485	NA	

Abbreviations: FIGO, International Federation of Gynecology and Obstetrics; CI, confidence interval; NA, not applicable.

**Table 3:** Univariate and multivariate analyses of disease-free survival in ovarian cancer patients

	Univariate analysis		Multivariate analysis	
Variables	Hazard ratio [95%CI]	*p* value	Hazard ratio [95%CI]	*p* value
Age (> 50)	1.62 [1.05–2.48]	0.028	1.46 [0.92–2.30]	0.108
FIGO stage (III/IV)	6.30 [2.90–13.68]	< 0.001	4.61 [2.10–10.13]	< 0.001
Cell type (non-serous)	0.37 [0.22–0.64]	< 0.001	0.63 [0.36–1.12]	0.119
Grade (poor)	1.86 [1.20–2.88]	0.006	1.73 [1.09–2.73]	0.019
CA125 (> 35 U/ml)	1.96 [0.94–4.05]	0.071	NA	
FILIP1L-	2.42 [1.49–3.93]	< 0.001	2.03 [1.21–3.41]	0.008
Beta-catenin+	1.32 [0.87–2.00]	0.192	NA	
SLUG+	1.55 [1.01–2.38]	0.044	1.89 [1.20–2.95]	0.006
FILIP1L-/Beta-catenin+	3.88 [1.78–8.46]	0.001	3.42 [1.53–7.64]	0.003
FILIP1L-/SLUG+	2.41 [1.34–4.34]	0.003	2.52 [1.36–4.65]	0.003
Beta-catenin+/SLUG+	1.97 [1.09–3.56]	0.024	2.76 [1.44–5.32]	0.002

Abbreviations: FIGO, International Federation of Gynecology and Obstetrics; CI, confidence interval; NA, not applicable.

### FILIP1L inhibits ovarian cancer metastases

**Figure 2:**
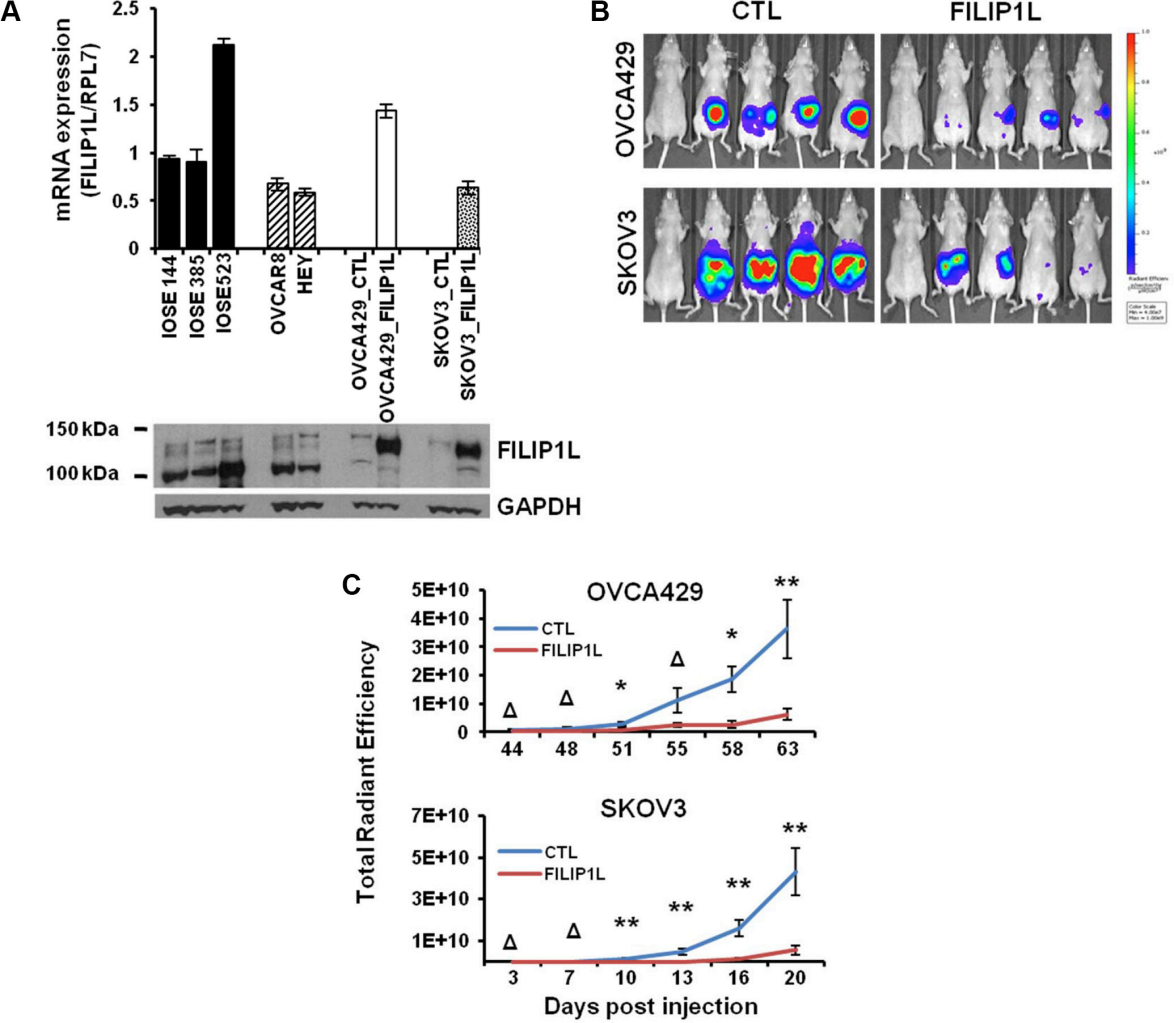
The effect of FILIP1L expression on the metastatic potential of ovarian cancer cell lines in a mouse model (**A**) *FILIP1L* mRNA levels were determined by qRT-PCR in immortalized normal IOSE cell lines and ovarian cancer cell lines that express high FILIP1L (OVCAR8 and HEY). Also shown are low FILIP1L-expressing OVCA429 and SKOV3 ovarian cancer cells, and their FILIP1L+ derivatives. The *y* axis represents *FILIP1L* mRNA expression standardized for the housekeeping gene *RPL7*. The result is an average of three independent experiments. Immunoblot analysis for FILIP1L in ovarian cells is also shown. Note that the FILIP1L band in FILIP1L+ OVCA429 and SKOV3 cells migrates slower than the other untransfected cells because it is expressed as a fusion protein with GFP. GAPDH blot is shown as the loading control. (**B**) OVCA429 (10^6^) and SKOV3 (5 × 10^5^) (CTL) and their FILIP1L+ derivatives (FILIP1L) were orthotopically injected into the ovaries of Nude mice. Development of peritoneal metastasis was monitored by IVIS imaging, every 3–4 days for a total of 63 and 20 days for OVCA429 and SKOV3, respectively. Representative images at the time of sacrifice are shown. The first mouse in each series is the negative control. (**C**) Time course of quantified metastatic tumor growth (8 mice per group). The *y* axis represents total radiant efficiency ([photons/s]/[μW/cm^2^]) emitted by iRFP. *,** and Δ indicate *P* < 0.05, *P* < 0.01 and NS, respectively. The result is representative of two independent experiments.

Having shown in clinical samples that FILIP1L expression decreases with tumor progression, we tested whether or not re-expression of FILIP1L could inhibit ovarian cancer metastases in an orthotopic mouse model. Two ovarian cancer cell lines, OVCA429 and SKOV3, were engineered to express FILIP1L at levels within the same order of magnitude as those in immortalized normal ovarian epithelial cells as shown by both mRNA and protein expression (Figure 2A), as well as expressing near-infrared fluorescent protein 720 (iRFP) [48]. Both control and FILIP1L+ clones were orthotopically injected into the ovaries of Nude mice and peritoneal metastatic spread was monitored by *in vivo* fluorescence imaging. FILIP1L+ derivatives developed markedly reduced peritoneal metastases compared to the parental cell lines (Figure 2B). As quantified by total radiant efficiency, FILIP1L inhibited 16- and 7-fold peritoneal metastases of OVCA429 and SKOV3, respectively (Figure 2C). As shown in Supplementary Figure S5, doxycycline-inducible FILIP1L+ clones from an ES2 cell line [39] also resulted in a significant reduction in peritoneal metastases, compared to the control demonstrating metastatic cancer spread into the peritoneum and the surface of visceral organs and the diaphragm. These results support our findings that FILIP1L is a potent tumor suppressor in ovarian cancer.

### FILIP1L knockdown induces chemoresistance in ovarian cancer cells

**Figure 3:**
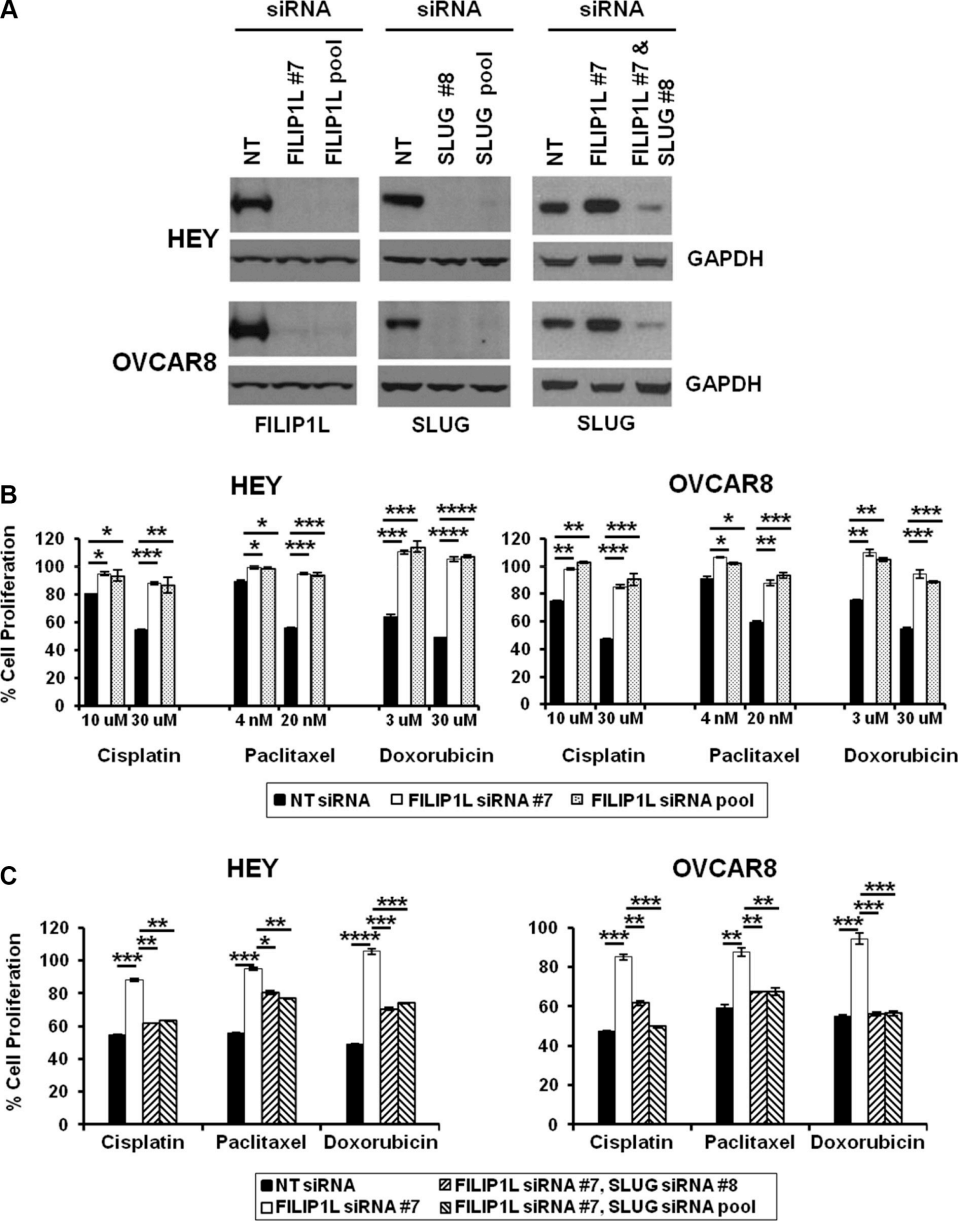
Effects of FILIP1L and an EMT activator on chemoresistance in ovarian cancer cells (**A**) Knockdown of FILIP1L and SLUG was achieved by transient transfection of indicated siRNA. Protein knockdown was shown by immunoblotting. GAPDH blot shown underneath of each blot is the loading control. The result is representative of two independent experiments. (**B**) Cells were treated with indicated siRNA for 2 days, and with chemotherapy agents of indicated concentration for an additional day. Cell proliferation was measured by WST1 incorporation quantified at OD_440-650nm_. The *y* axis represents cell proliferation in the presence of drug as a percentage of untreated control. (**C**) The same experimental procedures were used as described in section B with indicated siRNA treatment but only 30 μM cisplatin, 20 nM paclitaxel or 30 μM doxorubicin were used. *, **, *** and **** indicate *P* < 0.05, *P* < 0.01, *P* < 0.001 and *P* < 0.0001, respectively. The result is an average of three independent experiments.

As discussed, there is a direct association between metastatic potential, EMT and chemoresistance in cancer. Figure 1D and Supplementary Figure S1C show that in chemoresistant clinical samples, FILIP1L expression is decreased significantly, while SLUG expression is increased significantly. Thus, we explored the correlation between FILIP1L, SLUG and chemoresistance in ovarian cancer cells. To this end, we decreased FILIP1L and SLUG expression in two ovarian cancer cell lines from high-grade serous carcinoma [49], the relevant histosubtype for chemoresistance [1, 50] using siRNA transfection. FILIP1L and SLUG proteins were undetectable by immunoblot at day 2 post-siRNA transfection (Figure 3A). Suppression of FILIP1L increased the expression of SLUG, and SLUG expression was decreased by co-transfection of siRNAs for FILIP1L and SLUG (Figure 3A, third panel). Cells were treated with cisplatin, paclitaxel or doxorubicin following siRNA transfection. A cytotoxicity assay was used to confirm the markedly decreased ability of the three agents to kill ovarian cancer cells that no longer express FILIP1L (Figure 3B). In order to further prove cause-and-effect, we tested if SLUG knockdown can reverse the chemoresistant phenotype resulting from FILIP1L knockdown. Indeed, this phenotype was rescued by simultaneous knockdown of FILIP1L and SLUG (Figure 3C). Additionally, FILIP1L-high expressing cells such as HEY and OVCAR8 were more chemosensitive than FILIP1L-low expressing cells such as OVCA429 and SKOV3 (data not shown). Overall, our data indicate that chemoresistance is inversely correlated with FILIP1L expression and that FILIP1L may inhibit SLUG-mediated chemoresistance.

### FILIP1L regulates β-catenin degradation, the key transcriptional cofactor of WNT signaling

**Figure 4:**
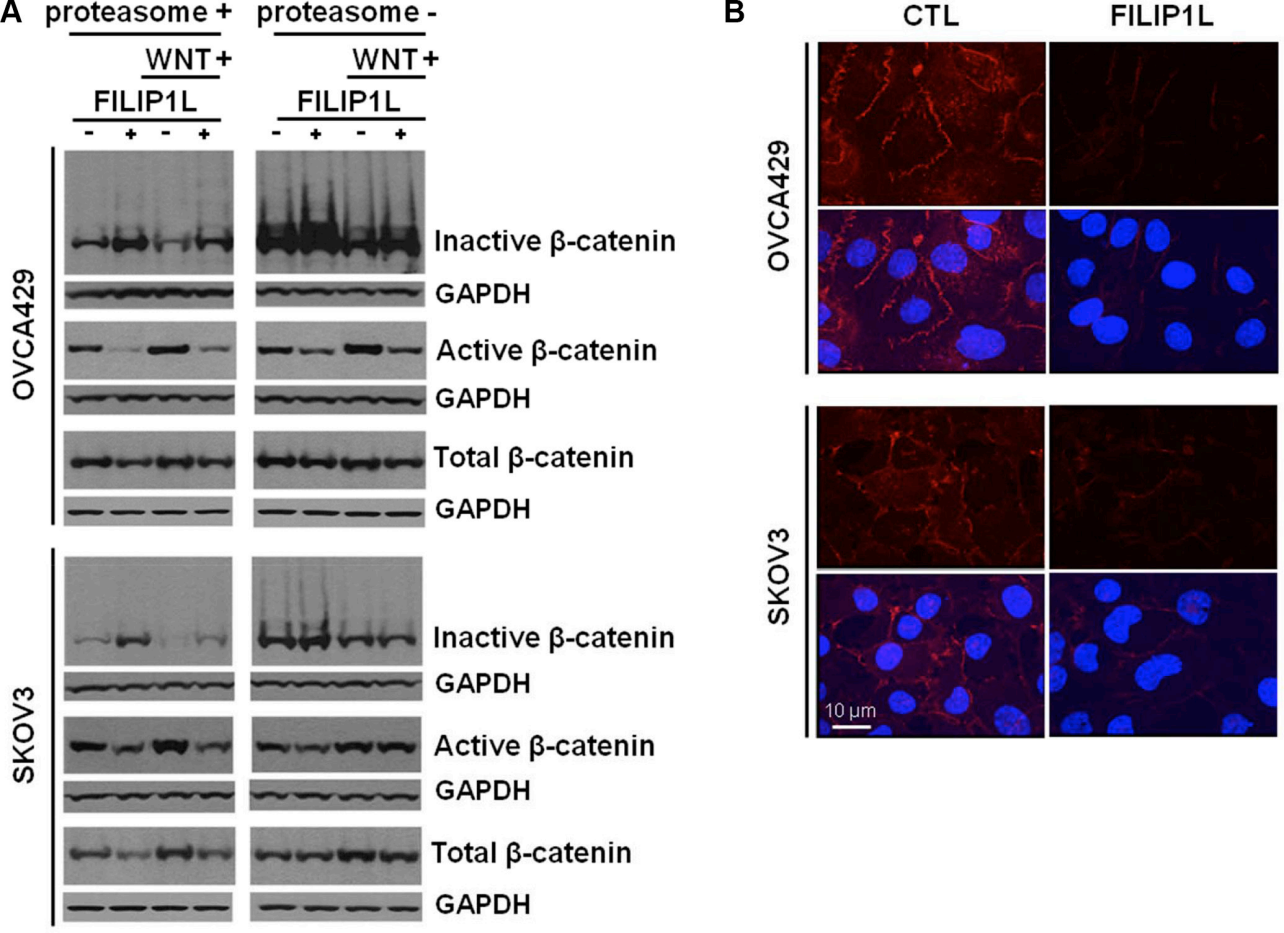
Effect of FILIP1L on β-catenin degradation by proteasomes (**A**) Parental and FILIP1L+ OVCA429 and SKOV3 clones were treated with solvent or 40 mM LiCl for 24 h (WNT+). Cell lysates were immunoblotted with antibodies against inactive phospho-β-catenin, active non-phospho-β-catenin, and total β-catenin. The experiment was also performed in the presence of 5 μM MG132 (proteasome-). GAPDH blot is shown as the loading control. (**B**) FILIP1L reduces active β-catenin. OVCA429 and SKOV3 clones were immunofluorescently stained for active β-catenin (red) and nuclei counterstained with DAPI (blue). Red only and merged images are shown. The result is representative of three independent experiments.

These experiments were directed at further elucidating the mechanism of action of FILIP1L. We previously showed that FILIP1L reduces nuclear β-catenin as well as transcriptional activation of canonical WNT-target genes. Additionally, we showed that FILIP1L localizes in the cytoplasm and centrosomes [39], recently identified as the proteolytic centers of the cell [51–54]. These observations prompted us to study the effects of FILIP1L on β-catenin availability. FILIP1L+ OVCA429 and SKOV3 clones were treated with a WNT activator (LiCl) [55], in the presence or absence of a proteasome inhibitor (MG132). We obtained similar results for activating WNT signaling using other agents including a WNT ligand WNT-3A (R&D systems) and GSK- 3β inhibitors such as SB-216763 (Sigma-Aldrich) and BIO (Calbiochem) (data not shown). Immunoblotting was used to detect levels of phosphorylated-β-catenin (inactive; destined for proteasomal degradation), non-phosphorylated β-catenin (active) and total β-catenin. Irrespective of WNT activation, the active β-catenin pool was decreased and the inactive pool increased in FILIP1L+ cells compared to parental cells (Figure 4A). The total β-catenin pool was relatively constant in all samples. When cells were treated with MG132, we observed increased inactive β-catenin levels. Since this phenomenon was independent of FILIP1L, it suggests that FILIP1L exerts its effects on β-catenin availability upstream of the proteasome. In addition, when FILIP1L was decreased using siRNA in FILIP1L-high expressing HEY and OVCAR8 cells, the phosphorylated β-catenin pool was decreased compared to the non-targeted control siRNA-transfected cells (Supplementary Figure S6). Next, we fractionated cells into nuclear, cytosolic and total cell lysates, and measured the levels of active and total β-catenin. As shown in Supplementary Figure S7, FILIP1L reduced the active β-catenin pool of nuclear and cytosolic fractions. These results were further confirmed by immunofluorescence staining. Active β-catenin localized in the cytosol, nucleus and cell membrane of control cells, and was significantly decreased in all of these compartments in FILIP1L-expressing cells (Figure 4B). FILIP1L expression and WNT signaling activation did not modulate β-catenin at the transcriptional level, as confirmed by qRT-PCR (data not shown). WNT ligand engagement results in phosphorylation of its receptor, LRP5/6. FILIP1L expression did not affect LRP5/6 phosphorylation (data not shown), which suggests that FILIP1L inhibits WNT signaling downstream of receptor activation. Collectively, these results suggest that FILIP1L decreases the pool of active β-catenin by targeting it for degradation.

### FILIP1L co-localizes with inactive β-catenin and proteasomes at the centrosomes

**Figure 5:**
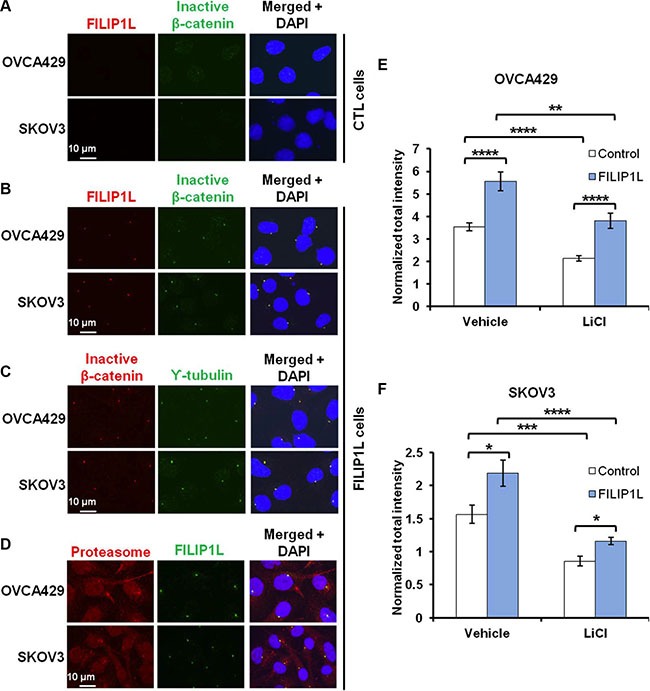
FILIP1L increases inactive β-catenin at centrosomes (**A–B**) OVCA429 and SKOV3 clones were immunofluorescently stained for FILIP1L (red) and inactive β-catenin (green). Staining is shown in either control (A) or FILIP1L-expressing (B) clones. (**C–D**) FILIP1L-expressing clones were immunofluorescently stained for inactive β-catenin (red) and γ-tubulin (green) (C) and for proteasome 19S-S7 subunit (red) and FILIP1L (green) (D). Nuclei were counterstained with DAPI (blue). Merged images are also shown. (**E**–**F**) The quantified data from 200-250 cells is also shown. *, **, *** and **** indicate *P* < 0.05, *P* < 0.01, *P* < 0.001 and *P* < 0.0001, respectively. The result is an average of three independent experiments.

Similar to FILIP1L [39], N-terminally phosphorylated β-catenin also localizes in centrosomes [56–58]. To investigate whether or not FILIP1L enhances degradation of phosphorylated β-catenin, we followed its cellular localization by immunofluorescence staining in OVCA429 and SKOV3 clones. As shown previously in ES2 cells [39], FILIP1L was distinctively localized to the centrosomes in FILIP1L+ clones from OVCA429 and SKOV3 cells, whereas endogenous FILIP1L was barely detectable in control clones (first columns of Figure 5A and 5B). Inactive β-catenin was present at higher levels in FILIP1L-expressing cells than in control cells (Figure 5A, 5B), and co-localized with FILIP1L (Figure 5B) and the centrosomal marker, γ-tubulin (Figure 5C). Moreover, FILIP1L co-localized with proteasomes in centrosomes (Figure 5D). Next, we quantified the amount of inactive β-catenin at the centrosomes. Inactive β-catenin was decreased following WNT activator (LiCl) treatment (Figure 5E, 5F). Irrespective of WNT activation, the inactive β-catenin pool was increased in FILIP1L+ cells compared to parental cells (Figure 5E, 5F). Together, our data strongly suggest that FILIP1L facilitates the degradation of phospho-β-catenin by proteasomes in centrosomes, thereby blocking WNT signaling.

### FILIP1L regulates EMT

**Figure 6:**
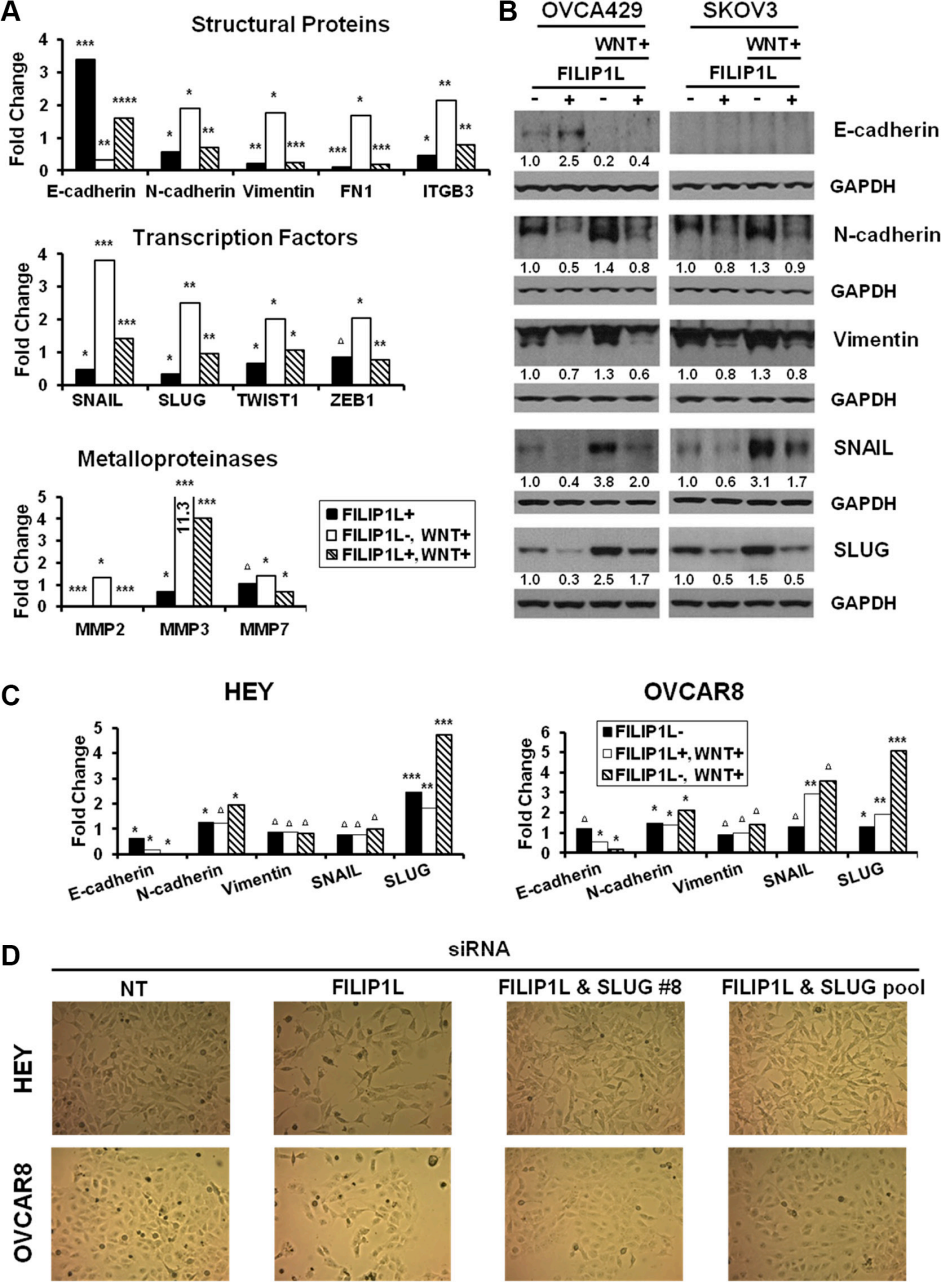
Regulation of EMT markers by WNT signaling and FILIP1L (**A**) Parental or FILIP1L+ OVCA429 clones were treated with solvent or 40 mM LiCl for 24 h (WNT+). mRNA levels of EMT markers were measured by qRT-PCR. The *y* axis represents fold change over the control (solvent-treated parental cells). Each value was also standardized for the housekeeping gene *GAPDH*. The result is an average of three independent experiments. (**B**) Immunoblot analysis for the indicated EMT markers from OVCA429 and SKOV3 clones treated with the same methods as in section A. GAPDH blot is shown as the loading control. Values indicate the quantified each protein amount normalized to the loading control GAPDH. Note that endogenous E-cadherin protein levels could not be detected due to low levels of expression in SKOV3 cells. The result is representative of three independent experiments. (**C**) HEY and OVCAR8 cells were treated with either non-targeting or *FILIP1L* siRNA, and with solvent or 40 mM LiCl for 24 h (WNT+). mRNA levels of EMT markers were measured as described in section A. The result is an average of three independent experiments. (**D**) HEY and OVCAR8 cells were treated with indicated siRNA for 2 days and cell morphology was imaged with light microscope. *, **, ***, **** and Δ indicate *P* < 0.05, *P* < 0.01, *P* < 0.001, *P* < 0.0001 and NS, respectively.

The WNT signaling induces EMT in ovarian cancer. [8, 12]. EMT results in repression of epithelial markers such as E-cadherin, and induction of mesenchymal markers, including adhesion molecules, metalloproteinases (MMPs) and transcriptional factors [16, 18]. A key feature of EMT is the loss of epithelial surface marker E-cadherin, and its down-regulation is often associated with transcriptional repression by EMT transcription factors such as SLUG [16, 18]. FILIP1L increases β-catenin degradation, which in turn inhibits WNT signaling. Our clinical and cellular data indicating potential associations between FILIP1L and SLUG led us to hypothesize that FILIP1L blocks EMT in ovarian cancer by virtue of decreasing the pool of active β-catenin. To unravel the possible connection between FILIP1L and EMT, the expression of EMT markers was examined following WNT signaling activation. First, we tested if low FILIP1L-expressing OVCA429 and SKOV3 cells acquired a more epithelial phenotype when engineered to express FILIP1L. Treatment of these cells with a WNT activator (LiCl) significantly decreased E-cadherin while increasing expression of mesenchymal markers. In contrast, FILIP1L+ clones are characterized by increased E-cadherin expression and a simultaneous decrease in levels of EMT transcription factors as well as mesenchymal markers (Figure 6A). Expression of FILIP1L largely abrogated the effects of LiCl-induced WNT signaling as evidenced by a reappearance of *E-cadherin* and repression of mesenchymal markers (Figure 6A). Protein expression of representative markers confirmed the inhibition of EMT by FILIP1L (Figure 6B). We also analyzed EMT marker expression in the primary tumors from orthotopic mouse models that we procured from the experiments shown in Figure 2B. As shown in Supplementary Figure S8, tumors from FILIP1L+ clones demonstrated decreased expression of mesenchymal markers as well as increased E-cadherin expression compared to those from control clones. Although the data from mouse tumors showed a trend towards EMT inhibition by FILIP1L, they were not completely correlated with those from cultured cells. For example, although expression of mesenchymal markers such as N-cadherin was significantly reduced, E-cadherin expression was not increased in FILIP1L+ tumors derived from OVCA429 cells. Among the mesenchymal markers, SLUG was consistently reduced in FILIP1L+ tumors from all three cell lines. We then confirmed the differential expression of adhesion markers at the protein levels using immunofluorescence staining, where N-cadherin expression was decreased significantly in FILIP1L+ OVCA429-tumors but E-cadherin expression was increased significantly in FILIP1L+ SKOV3 and ES2-tumors (Supplementary Figure S8).

Next, we tested if high FILIP1L-expressing HEY and OVCAR8 cells acquired a more mesenchymal phenotype when engineered to knockdown FILIP1L. FILIP1L knockdown in these cell lines resulted in increased expression of mesenchymal markers but decreased expression of E-cadherin (Figure 6C). Interestingly, among the mesenchymal markers, SLUG demonstrated a consistent increase following FILIP1L knockdown as well as a consistent induction following WNT activation in both cell lines. Increased expression of mesenchymal markers was accompanied by morphological changes where clustered epithelial-like cells became scattered spindle-shaped mesenchymal-like cells when FILIP1L was knocked down (Figure 6D). These morphological changes were reverted by simultaneous knockdown of FILIP1L and SLUG (Figure 6D), supporting the notion that FILIP1L exerts its anti-tumor activity by suppressing SLUG-mediated EMT in ovarian cancer cells.

## DISCUSSION

High mortality rate in ovarian cancer is mainly due to the fact that most patients are diagnosed with advanced stage disease with metastatic spread and that the majority of them succumb to recurrent, chemoresistant tumors. WNT/β-catenin signaling and its downstream target pathway EMT play a key role in ovarian cancer progression and chemoresistance [7–9,25]. Our data support an important role for FILIP1L in the control of this process. FILIP1L facilitates β-catenin degradation, thereby down-regulating EMT in ovarian cancer cells. FILIP1L expression is significantly decreased in metastatic as well as in chemoresistant clinical samples. Further, FILIP1L expression is inversely correlated with stage and poor prognosis. While our studies implicate FILIP1L from a mechanistic perspective as an important regulator of β-catenin at the protein level, these observations may also provide an important utility for FILIP1L as a clinical biomarker. The results we have presented suggest that the utility of determining FILIP1L expression in patients' samples could provide important prognostic information as high levels correlate with a good prognosis and lower probability of recurrence in ovarian cancer. This could allow the selection of patients with high FILIP1L expressing tumors who would be able to avoid over-intensive chemotherapy and its associated toxicities while focusing on patients with low expressing FILIP1L tumors who would most benefit from intensive chemotherapy regimens. This observation needs to be confirmed in a larger prospective cohort of patients.

As shown in the present study, the tumor suppressor FILIP1L is down-regulated in metastatic and chemoresistant ovarian cancer patient samples. Epigenetic regulators of tumor suppressors, including DNA methylation, histone modification and micro (mi) RNAs play a prominent role in cancer recurrence [59– 66]. We previously demonstrated significantly increased methylation of FILIP1L promoter residues in a region that also comprises a CREB/ATF binding site. CpG methylation was on average 10-fold higher in ovarian cancer cell lines compared to primary cells, and almost 2-fold higher in invasive serous carcinomas compared to borderline tumors. Methylation was inversely correlated with FILIP1L mRNA and protein levels, which were restored by a DNA demethylating agent [40]. We noted that treating ovarian cancer cell lines with a histone deacetylase inhibitor did not change FILIP1L mRNA and protein levels in these cell lines, leading us to conclude that histone acetylation was not a major epigenetic regulator of FILIP1L. To date, no studies have documented miRNAs that regulate FILIP1L expression by binding to 3′ untranslated regions (UTR). Thus it will be an important next step to identify the regulatory mechanisms by which FILIP1L is down-regulated in metastatic and chemoresistant samples.

**Figure 7:**
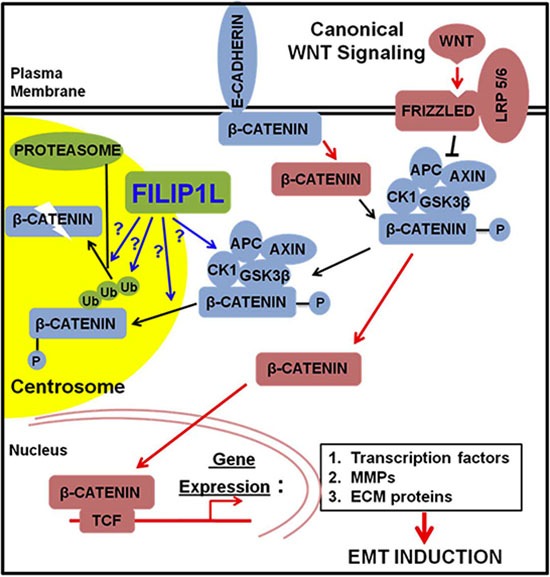
The proposed role of FILIP1L in inhibiting cancer cell invasion and metastasis through inhibition of WNT signaling Newly synthesized β-catenin is immobilized by E-cadherin at adherens junctions [10]. β-catenin can be released from the adherens junctions by downregulation of E-cadherin or by the activity of protein kinases. Free excess β-catenin is immediately phosphorylated by the destruction complex and thus marked for subsequent degradation. Canonical WNT signaling blocks the activity of the destruction complex resulting in increased levels of cytolasmic β-catenin, which is translocated to the nucleus. In the nucleus, β-catenin induces the expression of WNT target genes: 1) the transcription factors that repress the expression of *E-cadherin*; 2) MMPs that cleave E-cadherin as well as extracellular matrix (ECM); 3) the ECM and integrin molecules that favor cell-ECM adhesion. These events lead to induction of EMT. FILIP1L enhances β-catenin degradation in centrosomes possibly through: 1) inactivating the component(s) of β-catenin destruction complex, i.e. inactivating kinase activity of CK1 or GSK3β; 2) facilitating phospho-β-catenin recruitment into centrosomes; 3) facilitating polyubiquitination of phospho-β-catenin; 4) facilitating proteasome-mediated phospho-β-catenin degradation downstream of polyubiquitination. Downstream signaling of active β-catenin (via transcriptional regulation) is thus decreased.

In the present study, we show that FILIP1L colocalizes with phospho-β-catenin and proteasomes in centrosomes, the proteolytic center of the cell [51–54] and that phospho-β-catenin in centrosomes was reduced by FILIP1L following WNT signaling activation. These findings indicate that FILIP1L inhibits WNT signaling by facilitating degradation of phospho-β-catenin in centrosomes. The concept that centrosomal proteins antagonize WNT signaling in cancer cells establishes a new paradigm for tumor suppression. Figure 7 illustrates our proposed mechanism that FILIP1L facilitates phospho-β-catenin degradation through the ubiquitin-proteasome system in the centrosomes. This results in inhibition of β-catenin-dependent transcriptional regulation of various pathways that facilitate metastasis and chemoresistance, such as EMT.

We originally identified FILIP1L in angiogenesis setting. *FILIP1L* was shown to be specifically up-regulated in endothelial cells following the treatment of angiogenesis inhibitors [67, 68]. We then showed that FILIP1L expression in endothelial cells led to decreased cell migration and increased apoptosis, and that tumor vessel-expression of FILIP1L blocked *in vivo* tumor growth [42]. These data suggest that FILIP1L may be considered as an anti-angiogenic therapeutic. As anti-angiogenic strategy such as anti-VEGF therapy is another potential treatment in ovarian cancer, it potentiates the possibility of FILIP1L being a therapeutic target in ovarian cancer.

Ovarian carcinomas are heterogeneous, consisting of at least five different subtypes: high-grade serous (most prevalent), low-grade serous, mucinous, endometrioid and clear cell [50]. It was shown in ovarian cancer, mainly in endometrioid subtype that β-catenin mutants, resulting from activating and missense mutations, became resistant to proteosomal degradation [69]. Accumulating data indicate that although mutations in *β*-*catenin* and other genes in the WNT pathway are rare in other ovarian cancer subtypes than endometrioid, the WNT signaling plays a role in carcinogenesis of all subtypes of ovarian cancer [5– 7]. Increased β-catenin levels are commonly observed in different ovarian cancer subtypes and correlate with cancer stage and poor survival [6, 11–14]. This is consistent with our data where we have observed that the expression of FILIP1L, β-catenin and SLUG between serous and other subtypes are similar. These results are also supported by previous reports that have shown that the expression level of β-catenin and SLUG was similar between serous and other subtypes [14, 70, 71]. We speculate that FILIP1L may exert its tumor-suppressor activity by degrading increased β-catenin regardless of ovarian cancer subtype.

Although master EMT-regulating transcription factors SLUG and SNAIL [72] were shown to be directly associated with cisplatin and paclitaxel resistance in ovarian cancer [46, 47], SLUG but not SNAIL was consistently modulated by FILIP1L in our *in vitro*, *in vivo* and clinical specimen data. Induction of SLUG was consistently inhibited by FILIP1L following WNT signaling activation in several ovarian cancer cell lines tested. Simultaneous knockdown of SLUG reversed the chemoresistant as well as EMT phenotypes that had resulted from knockdown of FILIP1L. FILIP1L expression is decreased significantly, while SLUG expression is increased significantly, in chemoresistant clinical samples. In addition, the expression of FILIP1L was negatively correlated with the expression of SLUG in clinical samples, suggesting a link between FILIP1L and the SLUG-mediated EMT pathway in ovarian cancer. In the canonical WNT pathway, β-catenin is the crucial mediator responsible for the downstream transcriptional regulation [10]. The final transcriptional products are controlled by networking with other transcription factors in a context-dependent manner [10]. Our data suggest that expression of various EMT markers including EMT-regulating transcription factors can vary depending on cell lines, *in vitro* vs. *in vivo*, etc. Regardless, SLUG seems to be a consistent central downstream target for the FILIP1L-modulated WNT pathway in ovarian cancer. Future experiments employing ChIP-Seq and RNA-Seq analyses from FILIP1L-knockdown cells and tumors, will help identify key downstream target(s) of EMT.

In addition to activating EMT, β-catenin directly regulates the transcription of ATP-binding cassette (ABC) transporter genes such as ABCG2/BCRP and ABCB1/MDR1 in ovarian cancer [73,74]. ABC transporters have been implicated in chemoresistance and suggested as novel targets for treating cancer recurrence [75]. Isoliquiritigenin has been shown to augment chemosensitivity by targeting the β-catenin-mediated induction of ABCG2 in breast cancer [76]. Thus, it will be of interest to test if FILIP1L inhibits ABC transporter(s)-driven chemoresistance. However, as an immediate translational observation, determination of FILIP1L expression levels in patient tumors may be helpful in identifying those patients that are likely to be chemosensitive versus those that will be more resistant.

In summary, we have shown that FILIP1L expression is related with inhibition of metastases and chemoresistance, which is associated with down-regulation of EMT through β-catenin degradation. FILIP1L expression correlates with significantly improved overall survival, and is an independent prognostic factor for overall and disease-free survival in ovarian cancer. Further characterization of the mechanism of action of FILIP1L on chemosensitivity may help elucidate the role played by FILIP1L in ovarian cancer recurrence and result in the development of more effective treatment regimens. Since FILIP1L expression is inversely correlated with cancer invasiveness/aggressiveness in various other cancer types [39–41], these studies will also have an impact on treatment of other cancers.

## MATERIALS AND METHODS

### Tissue microarray and immunohistochemistry

A total of 64 normal epithelial tissues, 29 benign tumors (19 serous and 10 mucinous), 50 borderline tumors (21 serous, 24 mucinous, 1 clear cell and 4 mixed), 182 epithelial ovarian cancers (123 serous, 14 mucinous, 26 endometrioid, 10 clear cell, 7 transitional cell and 2 mixed) and 46 metastatic tumors were selected from patients who enrolled in the Gangnam Severance Hospital and the Korea Gynecologic Cancer Bank between 1996 and 2010. Some of the paraffin blocks were provided by the Korea Gynecologic Cancer Bank through the Bio and Medical Technology Development Program of the Ministry of Education, Science and Technology, Korea (NRF-2012M3A9B8021800). Ovarian cancer was staged according to the International Federation of Gynecology and Obstetrics (FIGO) staging system and graded according to the WHO grading system. The age of patients ranged from 22 to 80 years (average 52.3 years). Twelve patients presented with early recurrence during or within 6 months of initial treatment, which were grouped as a chemoresistant cases based on the clinical definition of chemoresistance to platinum in ovarian cancer.

Tissue microarrays (TMAs) were produced from formalin-fixed, paraffin-embedded tissues, and representative areas were meticulously selected from hematoxylin and eosin stained slides. Tissue cylinders of 1.0 mm diameter were extracted from selected areas of donor blocks and transplanted into recipient blocks using a tissue arrayer (Beecher Instruments). Tissue samples and medical records were obtained with informed consent of all patients and approval of the local research ethics committee (approval no. 2015–07–122; Seoul, South Korea). This study was additionally approved by the Office of Human Subjects Research at the National Institutes of Health.

For immunohistochemical staining, all paraffin sections were cut to 5 μm thickness, deparaffinized through xylene, and dehydrated through graded ethanol series. Antigen recovery was performed in heat-activated antigen retrieval buffer pH 6.0 (Dako). Endogenous peroxidase activity was blocked by addition of 3% H_2_O_2_. For FILIP1L, additional protein blocker (Dako) was applied. The slides were incubated at room temperature with antibodies against FILIP1L, β-catenin, SLUG and SNAIL. Antibodies are described in Supplementary Table S2. The antigen-antibody reaction was detected with EnVision+ Dual Link System-HRP and visualized with DAB+ (3, 3′-Diaminobenzidine; Dako).

The evaluation of immunostaining was carried out using computer-assisted image analyzing software version 4.5.1.324 (Visiopharm). Immunohistochemically stained slides were scanned using NanoZoomer 2.0 HT (Hamamatsu Photonics) at ×20 objective magnification (0.5 μm resolution), and captured digital images were then imported into the Visiopharm software. The threshold for size and shape of tumor cells was manually calibrated. Briefly, brown-colored (DAB-stained) and blue-colored (hematoxylin-stained) cells were separated spectrally. A brown staining intensity (0 = negative, 1 = weak, 2 = moderate and 3 = strong) was obtained using a predefined algorithm and optimized settings. The final histoscore was calculated by multiplying the intensity and percentage of staining resulting in score of 0 to 300 as described previously [77, 78]. The cut-off value of histoscore for discriminating between low and high expression was determined through receiver operating characteristic (ROC) analysis as described previously [79, 80].

### Cell culture and development of stable clones expressing FILIP1L

Human ovarian cancer cell lines ES2 and SKOV3 were purchased from American Type Culture Collection (ATCC) (Manassas). OVCA429 was provided by Dr. Barbara Vanderhyden (University of Ottawa), and HEY and OVCAR8 were provided by Dr. Gloria Huang (Albert Einstein College of Medicine) as described previously [40]. STR profiling of these cell lines were performed in March 2016 by the Genomics Core Facility at Albert Einstein College of Medicine using the Geneprint 10 kit (Promega). Cell suspensions were spotted on FTA cards and processed with an ABI 3730 DNA Analyzer. The alleles were analyzed by GeneMarker Software (SoftGenetics) and the allele reports (Supplementary Table S1) were compared with the ATCC Database or the published data [81].

FILIP1L-expressing OVCA429 and SKOV3 clones were developed as follows. Cells were transfected with piRFP-720 vector [48] followed by Neomycin selection. Resistant cells were then transfected with p110-GFP alone (GeneCopoeia) or encoding FILIP1L-GFP, followed by the selection of both Zeocin and fluorescence-activated cell sorting (FACS) for GFP. FILIP1L expression was tested by both qRT-PCR and immunoblot.

### Ovarian orthotopic model and IVIS imaging

All use of vertebrate animals described in this study was conducted in accordance with NIH regulations and was approved by the Animal Use Committee of Albert Einstein College of Medicine. Control or FILIP1L clones from each cell lines were injected under the bursal membrane of ovary in 8 week-old female nude mice (National Cancer Institute). Experimental details were followed as described previously [39]. For ES2 cells, mice were sacrificed at day 19 after orthotopic injection and metastatic tumor growth was quantified by weighing macroscopic metastatic tumors throughout peritoneal organs. For OVCA429 and SKOV3 clones that express iRFP (infra-red fluorescent protein; [48]), peritoneal tumor growth was monitored by *in vivo* imaging system (IVIS; LI-COR) as recommended by the manufacturer. Far red fluorescence was measured with excitation at 710 nm and emission at 760 nm, and total radiant efficiency was calculated with LivingImage software (LI-COR).

### SiRNA transfection

ON-TARGETplus Non-Targeting siRNA Pool (D-001810-10), *FILIP1L* siRNA (#7 (J-019458-07) and SMARTpool (set of 4 sequences; L-019458-00)) and *SLUG* siRNA (#8 (J-017386-08) and SMARTpool (L-017386-00)) were purchased from Thermo Scientific. HEY and OVCAR8 cells were transfected with equimolar amounts of either non-Targeting or *FILIP1L* and/or *SLUG* siRNA using Dharmafect solution following the manufacturer's protocols (Thermo Scientific). After a 48 h transfection, the cell lysates were subjected to qRT-PCR and immunoblot analysis.

### Immunoblot

Experimental details were followed as described previously [39]. For the preparation of cell fractionation, NE-PER nuclear and cytoplasmic extraction kit (Thermo Scientific) was used. Protein quantification from immunoblot was performed using AlphaView SA software (ProteinSimple).

### Quantitative real-time RT-PCR

Total RNA preparation and qRT-PCR was performed as described previously [40]. The gene-specific primers used with SYBR Green reagent are described in Supplementary Table S2. ITGB3 primers were purchased from Qiagen.

### WST1 cell proliferation assay

SiRNA-transfected cells were seeded in 96-well plates, treated with cisplatin, paclitaxel and doxorubicin for 24 h and incubated with WST1 (Roche) for 2 h. Cell proliferation by WST1 incorporation was measured using a Synergy Mx microplate reader (Biotek).

### Immunofluorescence staining

Experimental details were followed as described previously [39]. Images were acquired by an AxioCam HRM camera (Yokogawa) at 63× objective magnification (*z* stack of 0.4 μm thickness) on a Spinning disc confocal microscope (Zeiss; Observer Z1). Acquired images were then analyzed by AxioVision LE software (Zeiss). To quantify inactive β-catenin-, E-cadherin- or N-cadherin-positive signals, Cell Profiler software [82] was used as described previously [39].

### Statistical analysis

Statistical analysis for tissue microarray data was performed using R software package (version 3.1.2). The Mann-Whitney *U-test* was used to compare the protein expressions between each group. Analysis of the Spearman's rho coefficient was used to assess correlations between parameters. Survival distributions were estimated using the Kaplan-Meier method with the log-rank test. A Cox proportional hazards model was created to identify independent predictors of survivals. For the rest of experiments, data are presented as the mean ± SEM for the indicated number of separate experiments. Statistical analyses were performed using a two-tailed Student's *t* test (GraphPad Prism 3.0), and differences were considered statistically significant at *P* < 0.05.

## SUPPLEMENTARY MATERIALS TABLES AND FIGURES


