# Supplementary material for: Reduced expression of FILIP1L, a novel WNT pathway inhibitor, is associated with poor survival, progression and chemoresistance in ovarian cancer
Source: Oncotarget. 2016 Oct 20;7(47):77052–70. doi: 10.18632/oncotarget.12784 (PMC5340232; doi:10.18632/oncotarget.12784)
Supplement: Supplementary file 1 [file oncotarget-07-77052-s001.pdf]

Reduced expression of FILIP1L, a novel WNT pathway inhibitor, is associated with poor survival, progression and chemoresistance in ovarian cancer

Supplementary Materials

Supplementary Table S1: Summary of STR profiles of ovarian cancer cell lines used in this study

		TH01		D21S11		D5S818		D13S317		D7S820		D16S539		CSF1PO		AMEL		vWA		TPOX	
1	GM_GM0777_A05_SKL_ES2_10DIL_024.fsa	9.3	9.3	32.2	33.2	11	13	11	11	11	11	11	13	10	15	X	X	16	17	8	12
	100% Match to ATCC #CRL-1978																				
2	GM_GM0777_B05_SKL_OVCA429_10DIL_023.fsa	9	9	30	30	11	12	7	12	11	12	12	12	12	13	X	X	16	18	9	11
	100% Match to Korch et al. (PMID: 22710073)																				
3	GM_GM0777_C05_SKL_SKOV3_10DIL_022.fsa	9	9.3	30	31.2	11	11	8	11	13	14	12	12	11	11	X	X	18[a]	18	8	11
	92.86% Match to ATCC #HTB-77																				
4	GM_GM0777_D05_SKL_HEY_10DIL_018.fsa	8	9.3	30	30	11	12	11	11	12	12	8	12	10	11	X	X	16	17	11	11
	100% Match to Korch et al. (PMID: 22710073)																				
5	GM_GM0777_E05_SKL_OVCAR8_10DIL_017.fsa	7	7	28	28	12	12	12	12	12	12	13	13	11	11	X	X	17[a]	17	8	8
	92.86% Match to Korch et al. (PMID: 22710073)																				

[a]These numbers indicate mismatched alleles.

Supplementary Table S2: Summary of antibodies and qRT-PCR primers used in this study

Antibody	Source	Application
anti-FILIP1L	Aviva	IHC, IB
glyceraldehyde-3-phosphate dehydrogenase (GAPDH)	Millipore	IB
E-cadherin	BD Biosciences	IB, IF
N-cadherin	BD Biosciences	IB, IF
SNAIL	Novus Biologicals	IHC
SNAIL	Thermo Scientific	IB
β-catenin (phospho (Ser33/37/Thr41))	Cell Signaling	IB, IF
β-catenin (non-phospho)	Cell Signaling	IHC, IB, IF
β-catenin (total)	Cell Signaling	IB
SLUG	Abcam	IHC
SLUG	Cell Signaling	IB
vimentin	Cell Signaling	IB
lamin B1	Cell Signaling	IB
γ-tubulin	Sigma Aldrich	IF
proteasome 19S-S7 subunit	Abcam	IF

IHC: immunohistochemistry, IB: immunoblot, IF: immunofluorescence.

Gene (official symbol)	Forward primer	Reverse primer
E-CDH	GGAATCCAAGCAGAATTGC	TATGTGGCAATGCGTTCTCTATCCA
N-CDH	CTACAGACATGGAAGGCAATCC	CACTGATTCTGTACACTGCGTTC
VIM	GGTGGACCAGCTAACCAACGA	TCAAGGTCAAGACGTGCCAGA
FN1	GGGAGCCTCGAAGAGCAAG	AACCGGGCTTGCTTTGAC
SNAIL	CTTCCAGCAGCCCTACGAC	CGGTGGGGTTGAGGATCT
SLUG	GAGCATACAGCCCCATCACT	AGGAGGTGTCAGATGGAGGA
TWIST1	GGAGTCCGCAGTCTTACGAG	CCAGCTTGAGGGTCTGAATC
ZEB1	GCTGCCAATAAGCAAACGAT	CCATTTGGCTGGATCACTTT
MMP2	GCTGGCTGCCTTAGAACCTTTC	GAACCATCACTATGTGGGCTGAGA
MMP3	GAAGAGAAATTCCATGGAGCCAGG	AGAAATAAAAGAACCCAAATTCTTCAAAAACA
MMP7	GGGACATTCCTCTGATCCTAATGC	GAATTACTTCTCTTTCCATATAGTTTCTGAATGC
GAPDH	TGCACCACCAACTGCTTAG	AGAGGCAGGGATGATGTTC

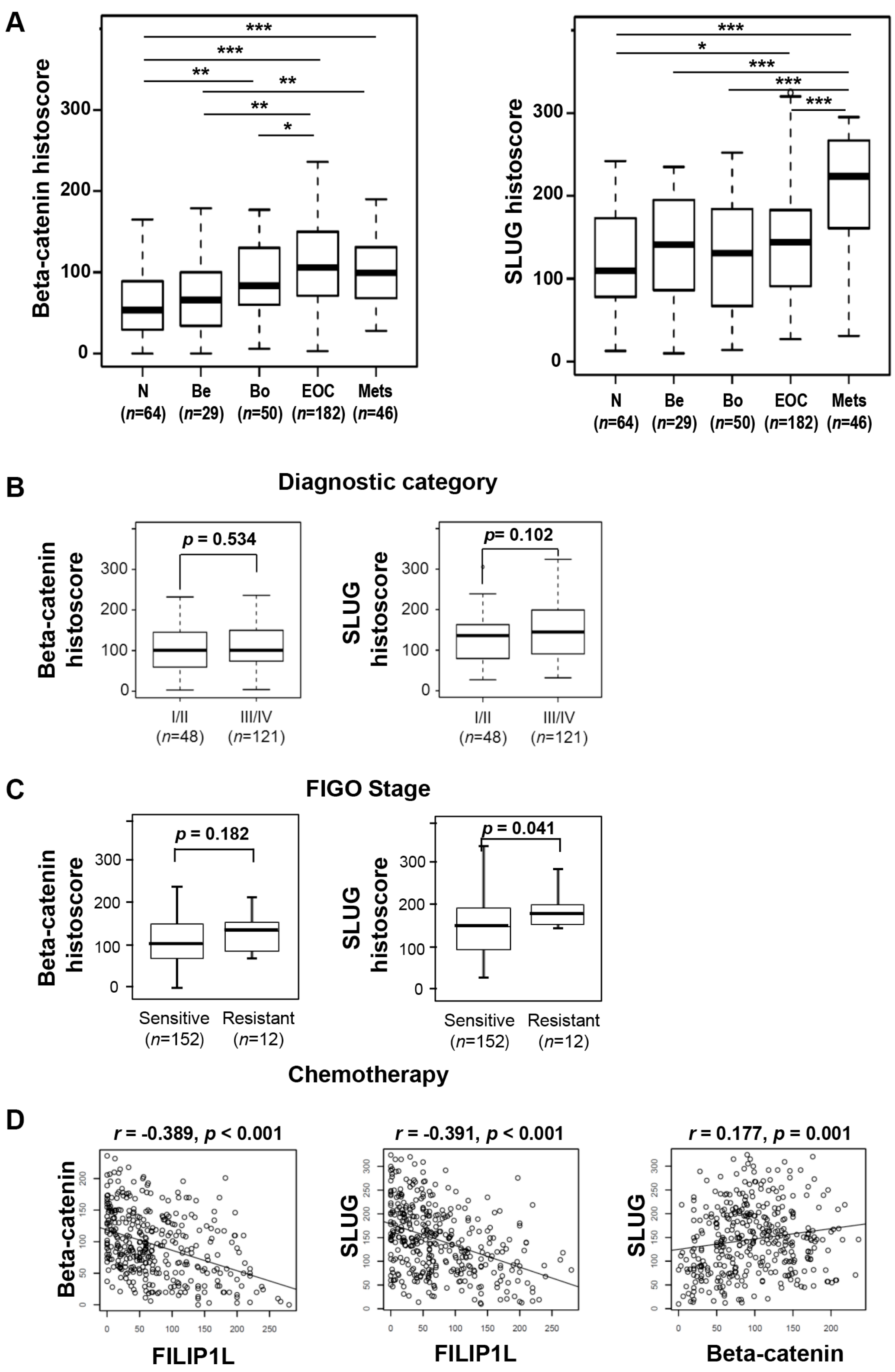
A
Beta-catenin histoscore
SLUG histoscore
N (n=64)
Be (n=29)
Bo (n=50)
EOC (n=182)
Mets (n=46)
B
Diagnostic category
p = 0.534
p= 0.102
I/II (n=48)
III/IV (n=121)
C
FIGO Stage
p = 0.182
p = 0.041
Sensitive (n=152)
Resistant (n=12)
Chemotherapy
D
r = -0.389, p < 0.001
r = -0.391, p < 0.001
r = 0.177, p = 0.001
Beta-catenin
SLUG
FILIP1L
Beta-catenin

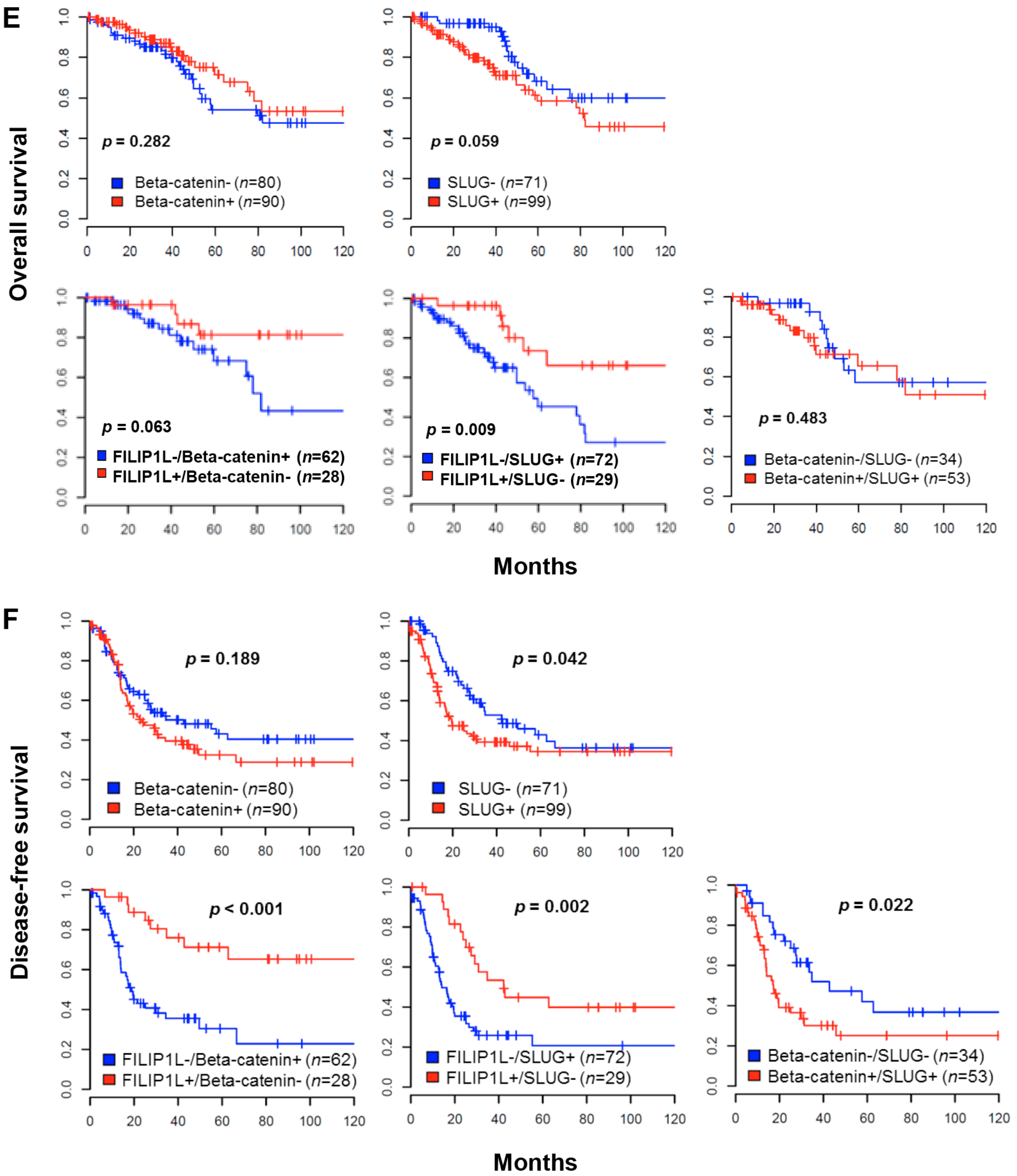

Supplementary Figure S1: Expression of FILIP1L, β-catenin and SLUG in normal and cancerous ovarian tissue. (**A**) Expression of β-catenin and SLUG was analyzed in specimens from normal (n = 64), benign (n = 29), borderline (n = 50), epithelial ovarian cancer (n = 123 for Serous type; n = 59 for other histologies) and metastasis (n = 46). *, ** and *** indicate $P < 0.05$, $P < 0.01$ and $P < 0.001$, respectively. B-C. Expression of β-catenin and SLUG was compared between specimens from FIGO stage I/II and stage III/IV ovarian cancers (**B**) as well as between specimens from chemo-sensitive and chemoresistant ovarian cancer patients (**C**). (**D**) Correlation between indicated protein expression in ovarian tissues was analyzed. (**E**–**F**) Overall survival (E) and Disease-free survival (F) of ovarian cancer patients according to the expression of FILIP1L, β-catenin, SLUG and their combination was analyzed in Kaplan–Meier plots. Histoscore of ≥ 75, ≥ 100 or ≥ 125 for FILIP1L, β-catenin or SLUG expression, respectively was considered positive.

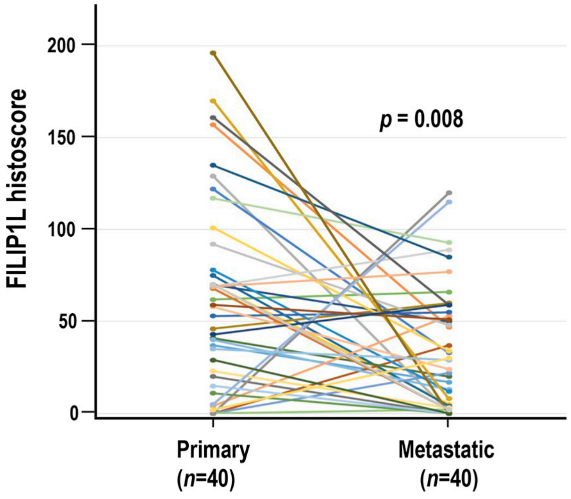

Supplementary Figure S2: Parallel coordinate plot analysis of FILIP1L expression in primary epithelial ovarian cancers and their matched metastatic tissues. *P* value was obtained from paired sample tests.

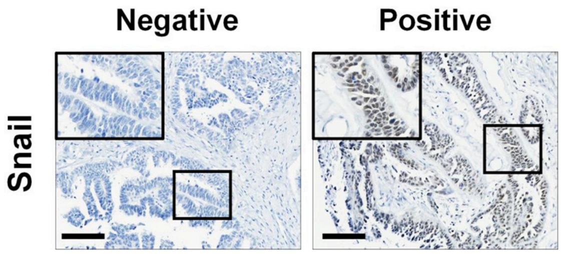

Supplementary Figure S3: Immunohistochemical staining of SNAIL in ovarian tissues. Representative images of human ovarian cancers with negative and positive SNAIL expression are shown. High magnification images are shown in inset. Scale bar shown is 100 µm.

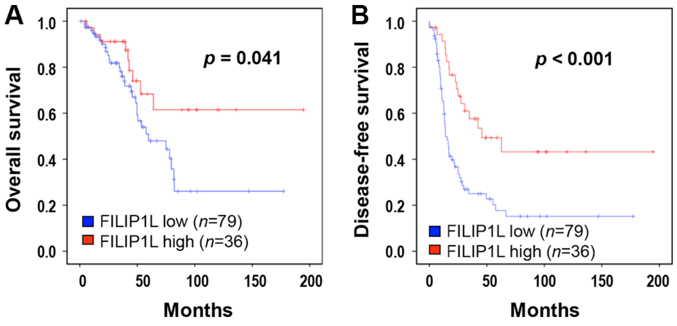

Supplementary Figure S4: Overall survival (A) and disease-free survival (B) of serous ovarian cancer patients based on FILIP1L expression was analyzed in Kaplan–Meier plots. Histoscore of ≥ 75 for FILIP1L expression was considered high.

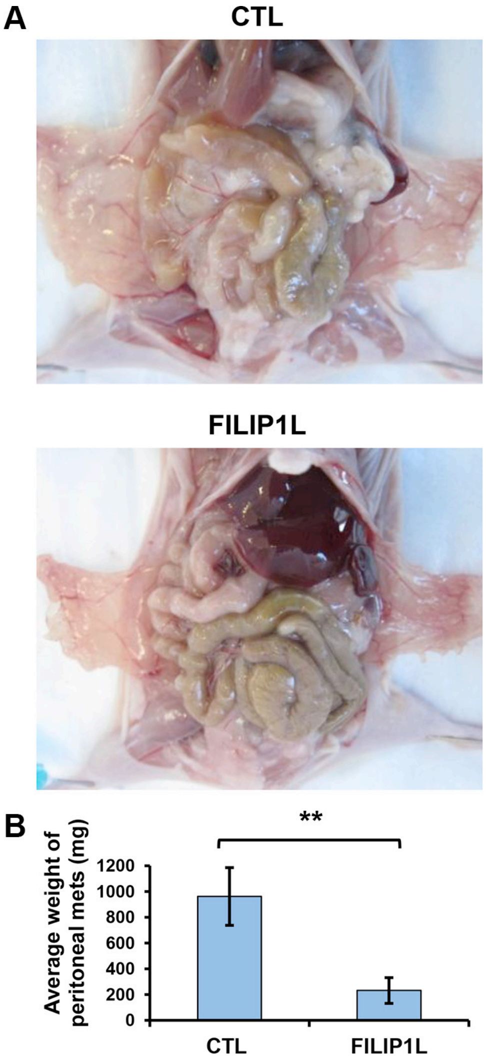

Supplementary Figure S5: The effect of FILIP1L expression on the metastatic potential of ES2 cells in a mouse model. Peritoneal metastasis from ES2 cells was examined at day 19 after orthotopic injection. Representative peritoneal images (**A**) and quantified metastatic tumor growth (**B**) are shown. The y axis represents the average metastatic tumor weight (8 mice per group). ** indicates $P < 0.01$.

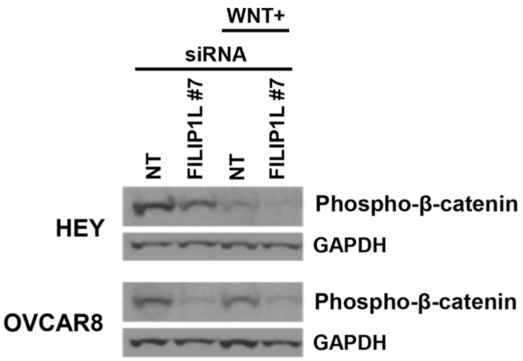

Supplementary Figure S6: FILIP1L knockdown reduces inactive β-catenin pool. FILIP1L-high expressing HEY and OVCAR8 cells were treated with indicated siRNA for 2 days, and with solvent or 40 mM LiCl (WNT+) for an additional day. Cell lysates were immunoblotted with antibodies against inactive phospho-β-catenin. GAPDH blot is shown as the loading control. The result is representative of two independent experiments.

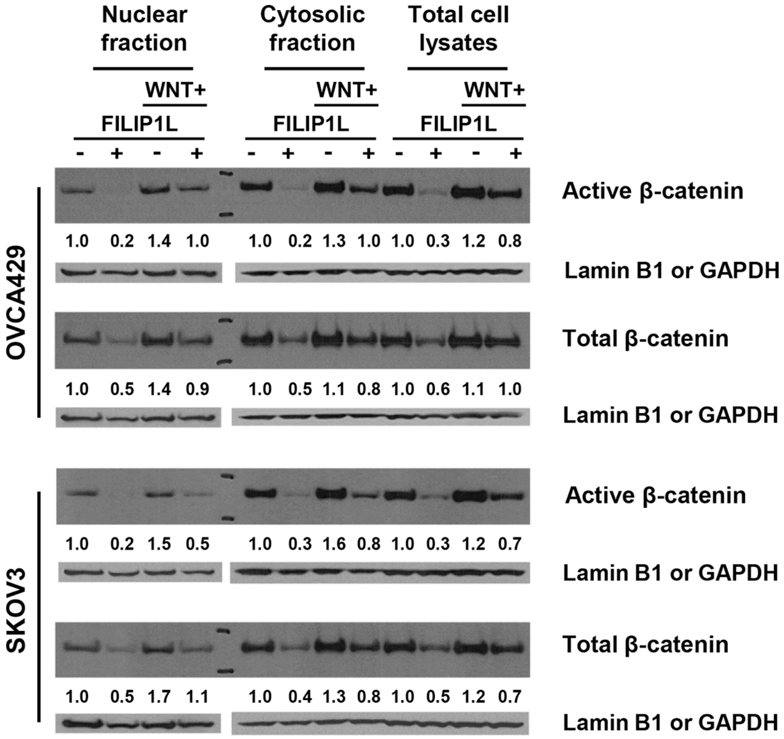

Supplementary Figure S7: FILIP1L reduces active β-catenin in the nucleus and cytosol. Parental and FILIP1L+ OVCA429 and SKOV3 clones were treated with solvent or 40 mM LiCl for 24 h (WNT+). Nuclear, cytosolic and total cell lysates were immunoblotted with antibodies against active non-phospho-β-catenin and total β-catenin. Lamin B1 (for nuclear) and GAPDH (for cytosolic and total) blot is shown as the loading control for cell lysates. Values indicate the quantified β-catenin amount normalized to the respective loading control, lamin B1 or GAPDH. The result is representative of three independent experiments.

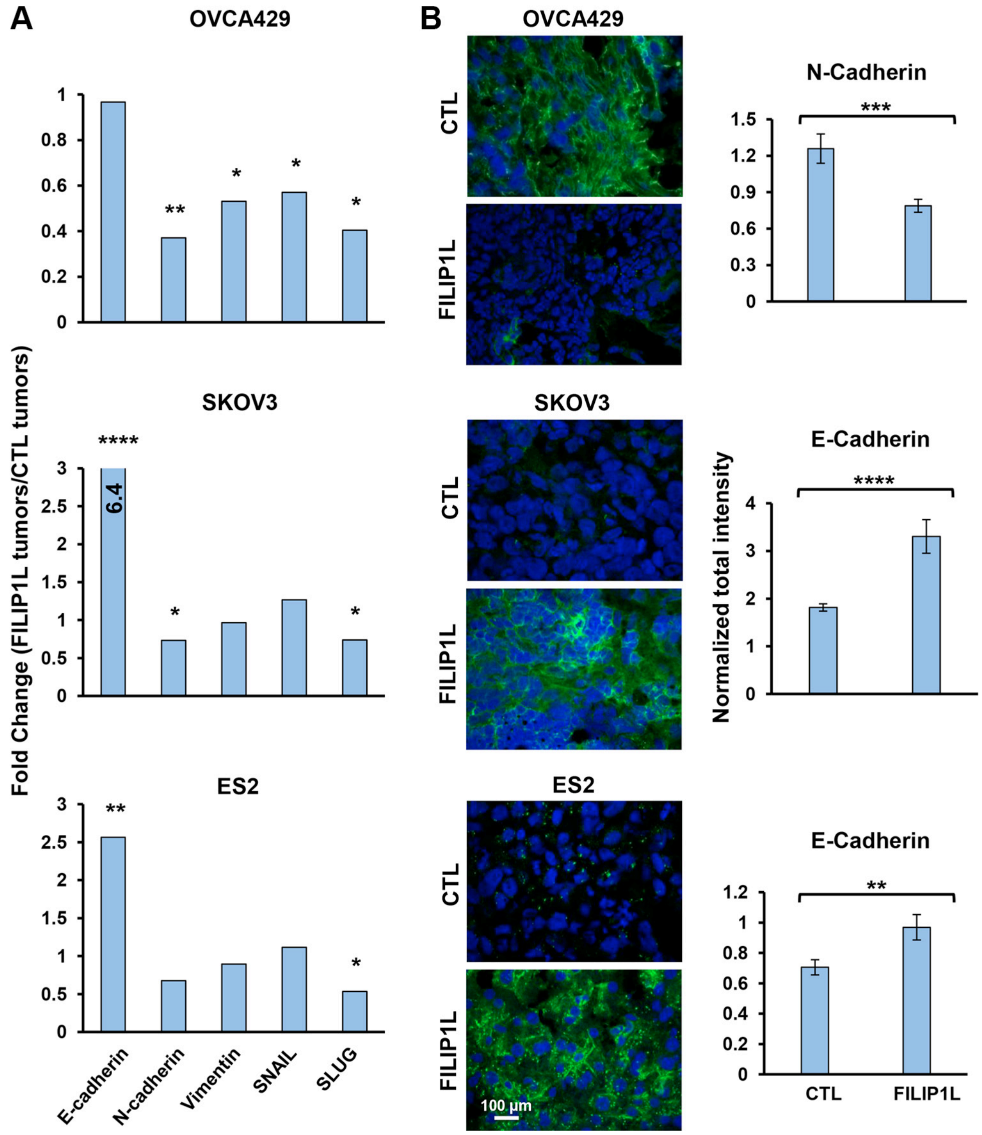

Supplementary Figure S8: FILIP1L inhibits expression of EMT markers in mouse primary tumors. (**A**) qRT-PCR analysis for EMT markers conducted on cDNA from FILIP1L clone-derived tumors. The y axis represents fold change of FILIP1L+ tumors (*n* = 6) over control tumors (*n* = 6), where each value was standardized with the housekeeping gene GAPDH. (**B**) Immunofluorescent staining of E-cadherin or N-cadherin (green) in FILIP1L clone-derived tumors. Nuclei counterstained with DAPI (blue). A merged image is shown. Shown at right is the quantified data from four random fields per tumor (*n* = 24). *, **, *** and **** indicate $P < 0.05$, $P < 0.01$, $P < 0.001$ and $P < 0.0001$, respectively.
